# Structural mechanism of anti-MHC-I antibody blocking of inhibitory NK cell receptors in tumor immunity

**DOI:** 10.21203/rs.3.rs-7133881/v1

**Published:** 2025-10-31

**Authors:** Jiansheng Jiang, Abir K. Panda, Kannan Natarajan, Haotian Lei, Shikha Sharma, Lisa F. Boyd, Reanne R. Towler, Sruthi Chempati, Javeed Ahmad, Abraham J. Morton, Zabrina C. Lang, Yi Sun, Nikolaos Sgourakis, Martin Meier-Schellersheim, Rick K. Huang, Ethan M. Shevach, David H. Margulies

**Affiliations:** 1 Molecular Biology Section, Laboratory of Immune System Biology, National Institute of Allergy and Infectious Diseases, National Institutes of Health, Bethesda, MD, 20892, USA; 2 Cellular Immunology Section, Laboratory of Immune System Biology, National Institute of Allergy and Infectious Diseases, National Institutes of Health, Bethesda, MD, 20892, USA; 3 Research Technology Branch, National Institute of Allergy and Infectious Diseases, National Institutes of Health, Bethesda, MD, 20892, USA; 4 Computational Systems Biology Section, Laboratory of Immune System Biology, National Institute of Allergy and Infectious Diseases, National Institutes of Health, Bethesda, MD, 20892, USA; 5 Fred and Pamela Buffett Cancer Center and Eppley Institute for Cancer Research, Omaha, NE, 68198, USA; 6 Laboratory of Cell Biology, Center for Cancer Research, National Cancer Institute, Bethesda, MD, 20892, USA; 7 Perelman School of Medicine, University of Pennsylvania

## Abstract

Anti-major histocompatibility complex class I (MHC-I) mAbs can stimulate immune responses to tumors and infections by blocking suppressive signals delivered via various immune inhibitory receptors. To understand such functions, we determined the structure of a highly cross-reactive anti-human MHC-I mAb, B1.23.2, in complex with the MHC-I molecule HLA-B*44:05 by both cryo-electron microscopy (cryo-EM) and X-ray crystallography. Structural models determined by the two methods were essentially identical revealing that B1.23.2 binds a conserved region on the α2_1_ helix that overlaps the killer immunoglobulin-like receptor (KIR) binding site. Structural comparison to KIR/HLA complexes reveals a mechanism by which B1.23.2 blocks inhibitory receptor interactions, leading to natural killer (NK) cell activation. B1.23.2 treatment of the human KLM-1 pancreatic cancer model in humanized (NSG-IL15) mice provides evidence of suppression of tumor growth. Such anti-MHC-I mAb that block inhibitory KIR/HLA interactions may prove useful for tumor immunotherapy.

MHC-I molecules, expressed on virtually all vertebrate somatic cells, play crucial roles in determining immunological and inflammatory responses to cancerous transformation and infection^1^. Classical MHC-I molecules, designated HLA-A, -B, and -C in the human, bind peptides and display peptide/MHC-I (pMHC) complexes at the cell surface for recognition by activating or inhibitory receptors on natural killer (NK) cells, monocytes, and T cells^2^. In humans, receptors expressed on NK cells and monocytes include killer immunoglobulin-like receptors (KIR) and leukocyte immunoglobulin-like receptors (LILRs or LIRs, also known as immunoglobulin-like transcripts (ILTs))^3,4^. Although KIR/HLA interactions exhibit a wide range of peptide preferences^5,6^, and LILRs bind with little peptide discrimination^7,8^, these interactions contrast with the exquisite peptide and MHC-I specificity exhibited by clonotypically variable T cell receptors (TCR)^9,10^. MAbs to MHC-I have been used extensively to characterize the function and polymorphism of this family of cell surface molecules and may also activate immune responses to tumors and infections by blocking interactions with MHC-I binding inhibitory receptors^11–13^.

Natural Killer (NK) cells play an important role in innate immunity against cancer and virus-infected cells^14–16^. NK cell function is regulated by activation and inhibitory receptors. Inhibitory receptors, such as KIR2DL and KIR3DL in the human or Ly49A and Ly49C in the mouse, recognize classical MHC-I (HLA or H2) molecules^4,17,18^. Inhibition of NK activity through signals conveyed by MHC-I/inhibitory receptor interactions maintains homeostasis, while loss of such signals either through reduced expression of MHC-I on tumor cells (so-called “missing self”^19^) or by antibody masking^11,12,20^ leads to NK cell activation. Numerous X-ray crystal structures of various NK receptors (KIR, Ly49) or receptors expressed on a wide spectrum of immune cells (such as LILR) alone, or in complex with MHC-I, have refined our understanding of the nature of recognition by these innate immune surface molecules^18,21,22^. A variety of anti-receptor antibodies that block KIR/MHC-I or LILR/MHC-I inhibitory signals and thus activate innate immunity have been under development as cancer immunotherapies^23–27^, administered either alone or in combination with antibodies that relieve checkpoint inhibition^28^.

Functional studies of two pan anti-human-MHC-I mAbs DX17 and W6/32^13^ and X-ray crystal structures of these in complex with representative HLA ligands^13,29^ provided an understanding of their ability to block the binding and tonic inhibition contributed by the interaction of inhibitory receptors such as LILRB1 with MHC-I. We recently observed that another anti-human-MHC-I mAb, B1.23.2^30,31^, also binds a wide range of human HLA-A, -B, and -C molecules. Cross-blocking experiments suggest that B1.23.2 functions to inhibit KIR interactions with MHC-I^32,33^. Thus, its biological activity may mimic or be complementary to that of pan anti-MHC-I mAbs^13^.

With recent technical and computational improvements, cryo-EM has become a valuable tool for determining the structures of protein complexes^34^. However, obtaining high-resolution structures of asymmetric complexes of modest molecular weight (less than 200 kDa) remains challenging^35^. Although some cryo-EM structures (using rigid and highly symmetric oligomeric scaffolds) have been determined at atomic resolution^36,37^, dynamic flexible molecules (e.g. full-length antibodies) remain difficult^36,38^. Here, we successfully determined the cryo-EM structures of complexes of B1.23.2 Fab and a B1.23.2 mAb in complex with the human HLA-B*44:05, at 3.31 Å and 3.02 Å resolution, respectively, as well as an X-ray crystal structure of B1.23.2 Fab complexed with HLA-B*44:05. These structures definitively describe the B1.23.2/HLA-B*44:05 interface and provide a structural paradigm for the informed application of B1.23.2 to reverse the effects of immune inhibitory receptors such as KIR2DL and KIR3DL. Analysis of these structures in the context of previously studied KIR/HLA and LILR/HLA complexes suggested that B1.23.2 would induce activation of human NK cells leading to anti-tumor activity. We show that B1.23.2, engineered to eliminate potential complications due to Fc receptor interactions, stimulates proliferation and activation of human PBMC-derived NK cells in culture and can lead to control of tumor growth in NSG-IL15 mice. Such structure-guided treatment offers potential benefits of anti-MHC approaches for cancer immunotherapy.

## RESULTS

### Binding of B1.23.2 to HLA-A, -B and -C

**Extended Data Fig. 1 |:**
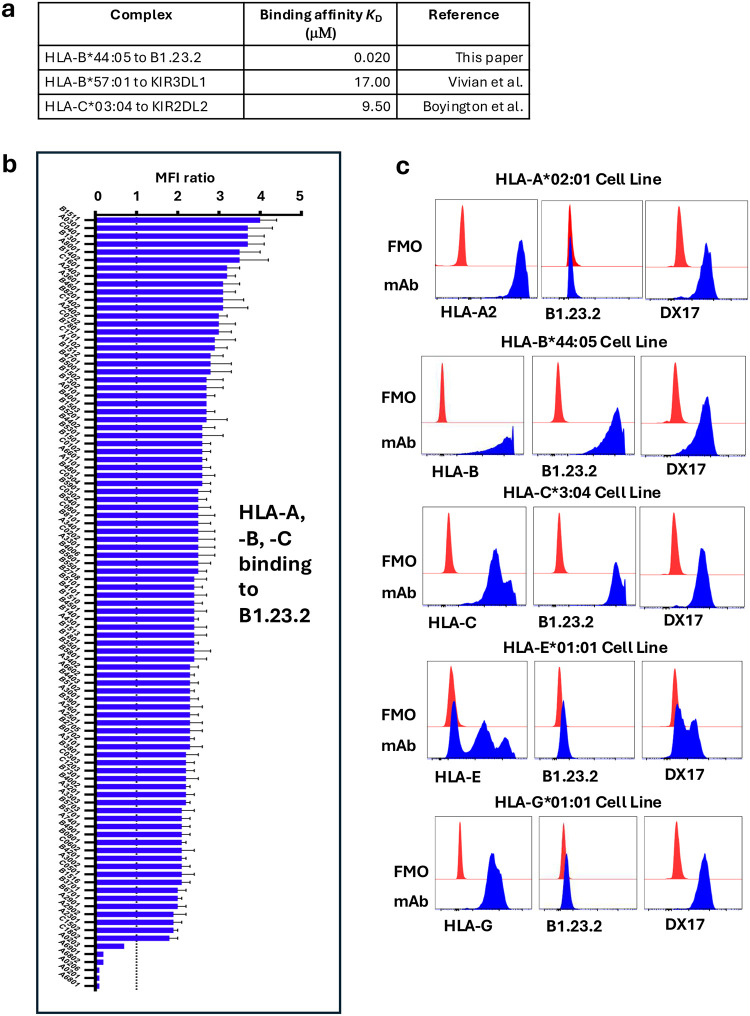
Binding affinity comparison, B1.23.2 specificity, and fluorescence staining of transfectants with B1.23.2 **a.** Comparison of binding affinity of B1.23.2 to HLA with KIRs that bind to various HLA. **b.** Binding of B1.23.2 to panel of Single Antigen Bead (SAB) screening as described in Methods. B1.23.2 binds all HLA-A, -B, -C relative to W6/32 except HLA-A*02:01, -A*02:03, -A*02:06, -A*68:01, -A*68:02 and -A*69:01. **c.** Fluorescence staining of transfectants expressing only the indicated HLA molecules. HeLa cells that were knocked out for endogenous expression of HLA-A, -B, and -C were transfected with cDNAs encoding the indicated HLA glycoproteins and then stained with allele-specific mAbs as described in Methods.

**Fig. 1 |:**
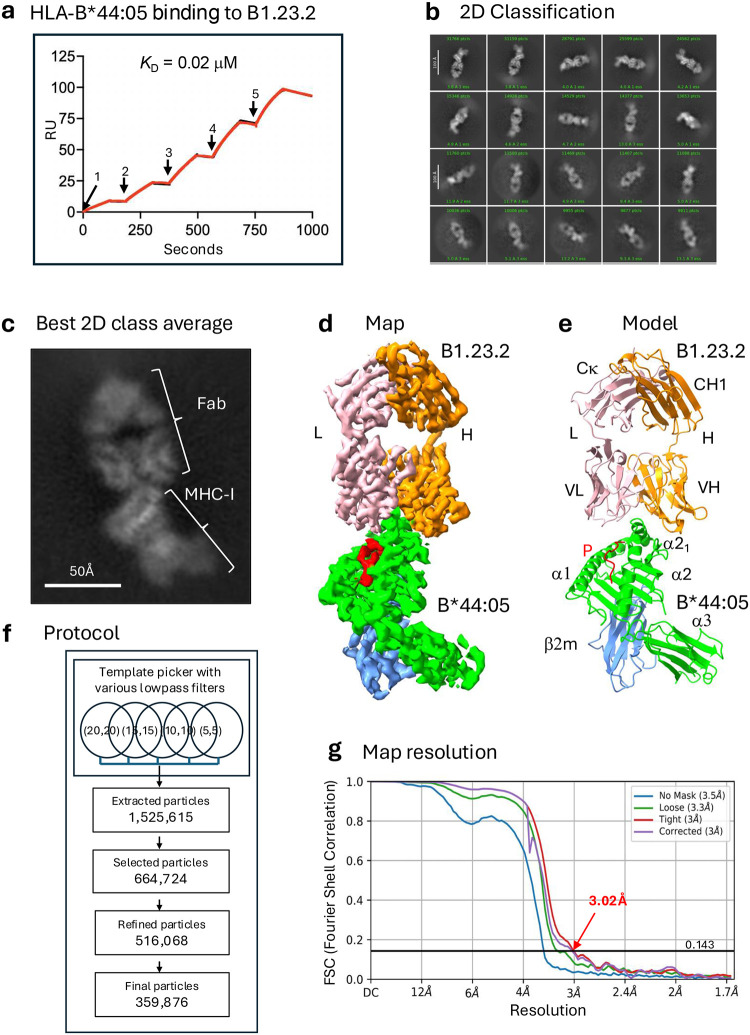
HLA-B*44:05 binding to B1.23.2 Ab, 2D classification of images, and overall structure. **a.** Binding of HLA-B*44:05 to B1.23.2 by SPR (see). Injection concentrations as indicated: 1=31nM, 2=62nM, 3=125nM, 4=250nM, and 5=500nM. **b.** 2D classification of cryo-EM images after several runs of particle picking. **c.** The best 2D class shows domains of Fab and MHC-I. **d.** Cryo-EM map of the complex. **e.** Refined model (PDB ID: 9D73) with the same colors indicating each domain and peptide. **f.** The protocol (see Extended Data Fig. 2) used in template picking and the number of resulting particles in each step. **g.** Map resolution as indicated by FSC (Fourier Shell Correlation) at 0.143 reveals 3.02 Å resolution (no mask = 3.5 Å).

Previous studies showed that B1.23.2 blocked the binding of recombinant KIR2DS1 and KIR2DL1 to HLA-C*04:01 transfectants^32^. In addition, B1.23.2 inhibits the interaction of a KIR2DL1 reporter cell with HLA-C*02:02/04:01^+^ fibroblasts and partially blocks a KIR2DS1 reporter^33^. We extended these experiments using an engineered, recombinant B1.23.2, which blocked the staining of human PBMC-derived CD14^+^ monocytes or of single HLA-B or -C transfectants by recombinant soluble KIR2DL2 and KIR2DL3, and by KIR3DL1^13^. Using surface plasmon resonance (SPR), we show that recombinant B1.23.2 binds to HLA-B*44:05 with a *K*_D_ = 0.02 μM (Fig. 1a), which is stronger than the range of affinities reported for KIR/HLA interactions (Extended Data Fig. 1a)^6,39,40^. B1.23.2, originally reported to react with an HLA-A, -B positive cell line, independent of the β_2_m light chain at a site distinct from W6/32^30^, binds a broad panel of different HLA molecules, including all HLA-B and HLA-C tested^31^. We evaluated the specificity of binding of the recombinant B1.23.2 mAb to a standard panel of HLA-A, -B, and -C allelomorphs (Extended Data Fig. 1b). Using the pan anti-HLA mAb W6/32 as a reference^41^, we observed that B1.23.2 binds all HLA-A, -B, and -C molecules tested with the exception of HLA-A*02:01, -A*02:03, -A*02:06, -A*68:01, -A*68:02, and -A*69:01. Consistent with these results, B1.23.2 stained cells individually transfected with HLA-B*44:05 and HLA-C*03:04, but failed to stain those expressing HLA-A*02:01, HLA-E*01:01, or HLA-G*01:01 (Extended Data Fig. 1c). Together, these results indicate that B1.23.2 blocks KIR2 and KIR3 binding to HLA, binds HLA with nanmolar affinity, and shows broad reactivity to HLA-A, -B, and -C allelomorphs.

### Cryo-EM structure of anti-MHC-I mAb B1.23.2 in complex with HLA-B*44:05

**Extended Data Fig. 2 |:**
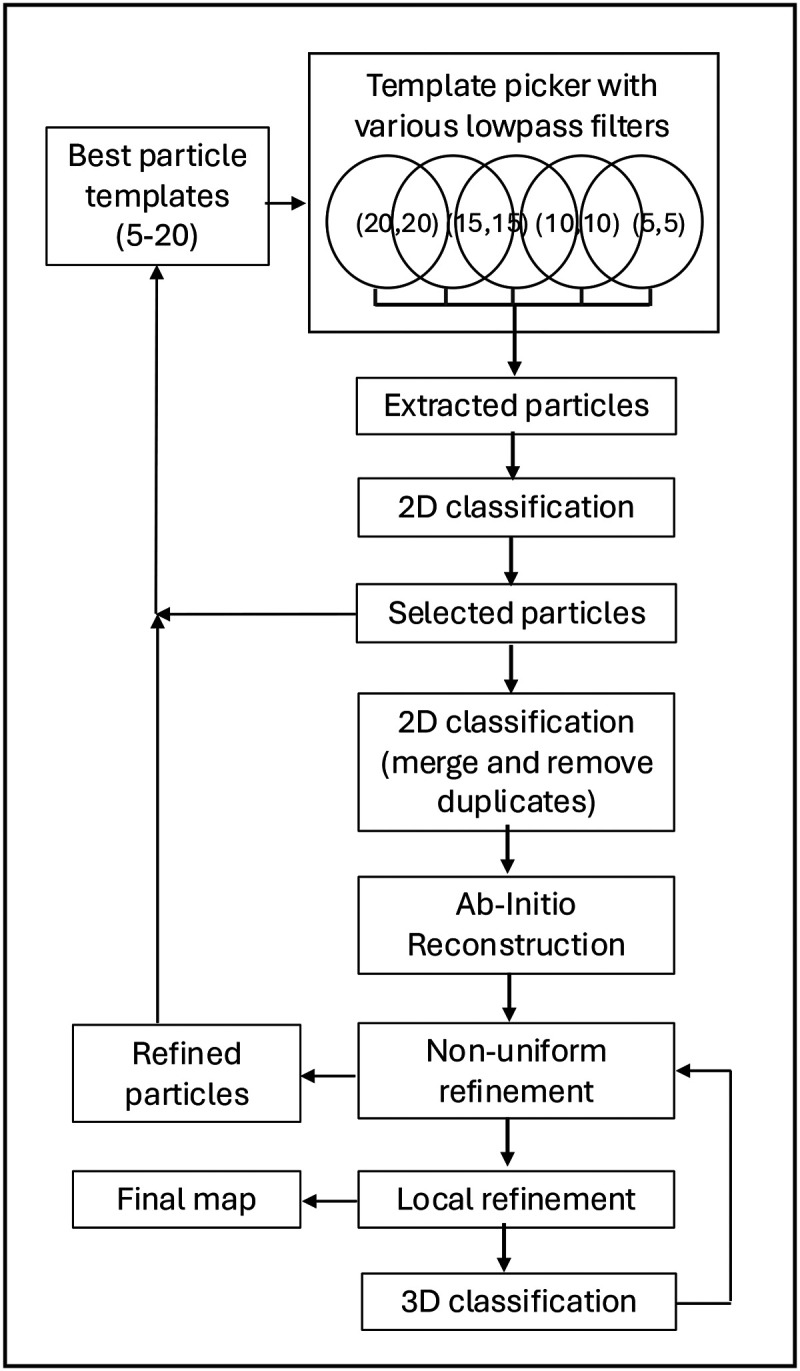
A protocol for map resolution improvement using cryoSPARC. In the Template picker, with various Lowpass filters (LPF) applied to the template particle and micrograph, multiple runs are submitted in parallel. Then, particles with individual 2D classification are extracted, and 2D classification is performed. Selected particles are iteratively cycled. A second round of 2D classification is performed with all previously selected particles, and duplicates are removed. In the ab initio reconstruction, following standard Non-uniform and local refinement, a 3D classification is applied to remove some low-resolution classes.

To understand how B1.23.2 blocks the binding of KIRs to their MHC ligands, we determined cryo-EM structures of the complex of B1.23.2 with HLA-B*44:05 using both a full-length engineered Ab and the Fab derived from the murine hybridoma. Complexes of B1.23.2 mAb with bacterially expressed and refolded recombinant peptide/HLA-B*44:05/β_2_m were produced, and cryo-EM images were collected as described in the Methods. Using cryoSPARC^42^, we developed a protocol that improved map resolution for small molecular weight nonglobular complexes (Extended Data Fig. 2). Table 1 summarizes the data collection, statistics, and validation for the two cryo-EM structures of B1.23.2 mAb/HLA-B*44:05 and B1.23.2 Fab/HLA-B*44:05 complexes as well as for the Fc region images collected from the B1.23.2 mAb/HLA-B*44:05 complex. (Amino acid sequences are presented in Extended Data Table 1).

The cryo-EM structures determined from both the full length B1.23.2 mAb and Fab complexes with HLA-B*44:05 were very similar. The resolution of the mAb-containing complex was slightly better, and we discuss this structure first. Fig.1b shows the 2D classification of particles of B1.23.2 mAb/B*44:05, revealing shapes clearly consistent with the expected Fab/HLA-B*44:05/β_2_m complex (Fig. 1c). We noticed multiple distinct images among the 2D classes – some appear as Fab complexed with MHC-I; others look like Fc alone. We did not observe individual particles representing full-length mAb, likely due to the flexibility of the hinge between Fab and Fc. The final refined map of the Fab/HLA images from the full-length mAb-containing sample (Fig. 1d) clearly showed all eight domains (V_H_, C_H1_, V_L_, Cκ of the mAb and α1, α2, α3, β2m of HLA-B*44:05) and the bound peptide. The model (Fig. 1e) that was fit to the map was refined and validated (Table 1). The cryo-EM map was based on 359,876 particles and resulted in a map of resolution 3.02 Å, as shown in Fig.1f,g.

**Fig. 2 |:**
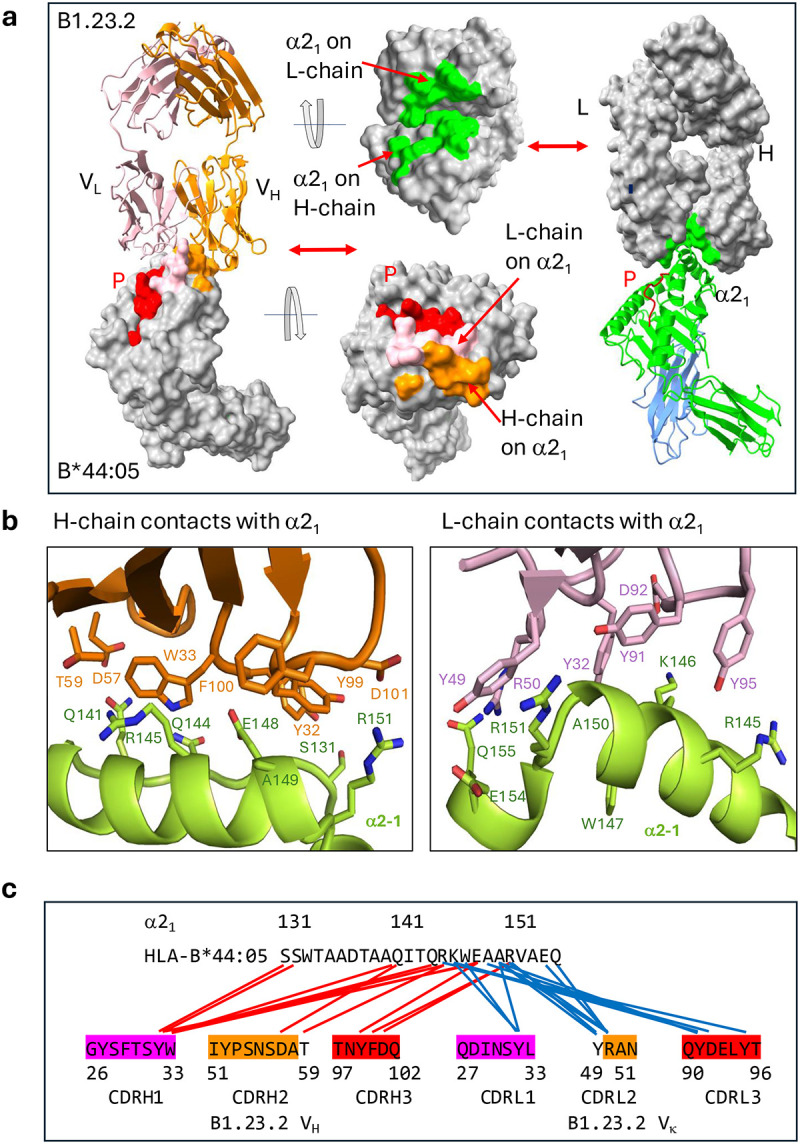
B1.23.2 interacts with HLA-B*44:05 through H and L chain CDR loop contacts. **a.** The footprints of B*44:05 on the surface of B1.23.2 are indicated as green, and the footprints of B1.23.2 on the surface of B*44:05 are shown as orange (heavy chain) and pink (light chain). The total Buried Surface Area (BSA) is 899 Å^2^. **b.** Details of the interaction of CDR loops of the light and the heavy chains. Both chains contact the a2_1_ helix (see Extended Data Table 2). **c.** A diagrammatic presentation of the contacts. The multiple contacts (from CDR loops of H or L chains) are aligned against the residues of the α2_1_ helix. Contact between HLA-B*44:05 S8 and L chain Y32 is not shown.

Analysis of the contacts at the B1.23.2/HLA-B*44:05 interface reveals that the major focus of the antibody V_H_ and V_L_ is the HLA α2_1_ helical segment. The Fab/HLA complex has a buried surface area (BSA) of 899 Å^2^ consistent with many Ab/protein Ag interfaces (Fig. 2a). Neither V_H_ nor V_L_ of B1.23.2 interacts with β_2_m. The details of the interactions are shown in Fig. 2b and Extended Data Table 2a and a contact map in Fig. 2c. The complementarity determining region (CDR) loops of V_H_ and V_L_ recognize the conserved residues on the α2_1_ helix of HLA-B*44:05. Interestingly, the entire α2_1_ helix of B*44:05 from Q141 to Q155 is buried tightly in a groove formed by V_H_ and V_L_ and constitutes the B1.23.2 epitope (Fig. 2a). K146 of HLA-B*44:05 is a major epitopic residue as it is bound by four residues (Y27, D87, Y86, and Y90) of V_L_ of B1.23.2 (Fig. 2b, right panel). Epitopic residues Q141, E148, A150 make multiple contacts with B1.23.2, and R145, R151, and A149 contact both V_H_ and V_L_ of B1.23.2 (Fig. 2c, Extended Data Table 2a). Overall, Y28, W29, and Y95 of V_H_, and Y27, Y44, and Y90 of V_L_ of B1.23.2 make the major contributions to the recognition of the α2_1_ helix of B44:05, emphasizing the general role that tyrosine and tryptophan play in antibody recognition^43^.

**Extended Data Fig. 3 |:**
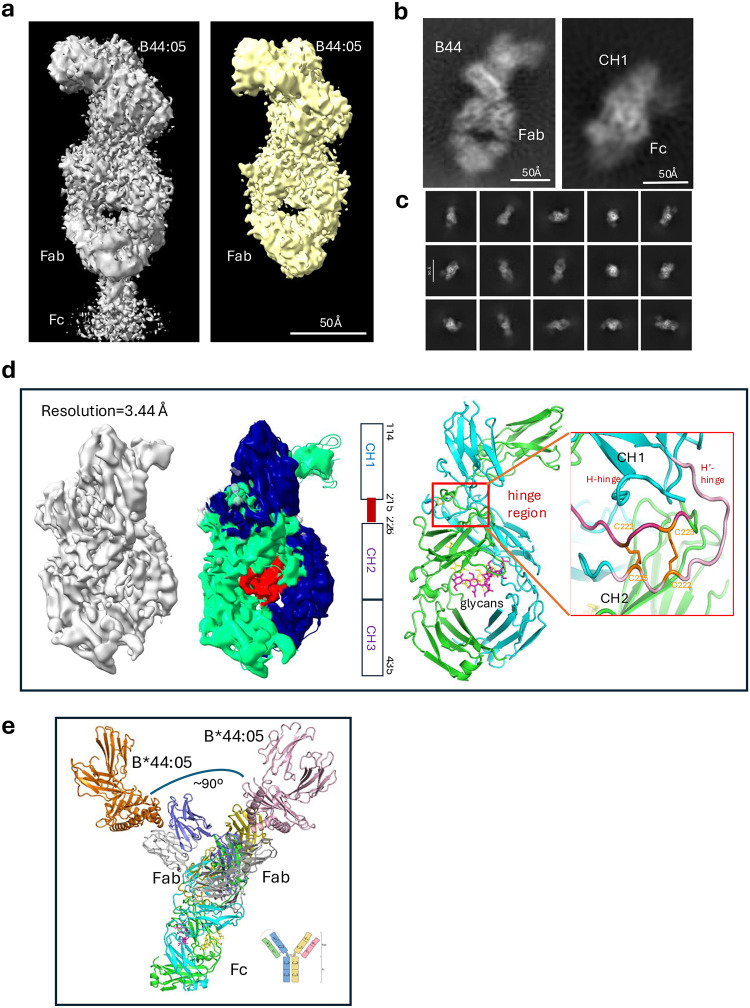
B1.23.2Ab analysis reveals Fc-like Domains. **a.** Comparison of the map of B1.23.2 mAb+B*44:05 and B1.23.2 Fab+B*44:05 at the same contour level (0.02 e/Å). **b.** Two types of particles were observed: Fab+B44-like and Fc-like. The Fc domain has an extension that might be part of the C_H1_ domain. **c.** 2D classes for Fc-like particles (selected representatives). **d.** The Fc-like map (3.44Å resolution with 251,084 particles) was fit to the model. Two H-chains are colored blue and cyan, and red indicates the glycans. The model presents the partial C_H1_ domain, the disordered hinge region, and two twisted C_H1_ domains; the hinge loops “cross-over” to form two disulfide bridges. The glycans are linked to N293 of the C_H2_ domain. **e.** A reconstructed model of full-length B1.23.2 with B*44:05 shows the hinge angle of the two Fabs to be 90° −100°; however, the light chain (gray) clashes at the hinge region.

Although we did not directly observe complete particles of full-length B1.23.2 with HLA-B*44:05 in a single stand-alone reconstruction, some additional density adjacent to the Fab fragment was observed. This density extends beyond the C_H1_ domain of the Fab in the full-length B1.23.2 sample (Extended Data Fig. 3a, left panel), which likely represents part of the C_H2_ region of the Fc. We observed multiple types of particles in the micrographs from the full-length B1.23.2 sample – both Fab/HLA-B*44:05-like and Fc-like (Extended Data Fig. 3b). The Fc-like particles accounted for less than 10% of the total. Extended Data Fig. 3c illustrates some of the 2D classes of the Fc-like particles. The length of the Fc-like particles in the 3D reconstructed map measures about 100 Å, including a part of the C_H1_ domain (Extended Data Fig. 3d). This map was refined (using non-uniform refinement with 251,084 particles) to a resolution of 3.44 Å. Model fitting was challenging, largely due to disorder in the hinge region connecting C_H1_ to C_H2_ (Extended Data Fig. 3d). Using an hIgG1 Fc domain model generated by AlphaFold 3^27^, we could fit part of the C_H1_ domain as well as C_H2_ and C_H3_ (Extended Data Fig. 3d, center). The two C_H1_ domains are twisted, and the hinge loops appear to form two “cross-over” disulfides^44,45^ at residues C222-C225 (Extended Data Fig. 3d, insert). Glycans linked through N293 of the C_H2_ domains were also identified. By overlaying the C_H1_ domains identified in the Fc maps with the extension seen on some Fab images, we constructed composite full-length models of B1.23.2 (Extended Data Fig. 3e) in which the angle between the two Fabs may vary from 90° to 100°. Light chain residues are seen to sterically compete with other density in the hinge region, suggestive of flexibility, mobility, and dynamics – precluding the detailed visualization of the whole B1.23.2 antibody.

### Cryo-EM structure of anti-MHC-I Fab of B1.23.2 in complex with HLA-B*44:05

**Extended Data Fig. 4 |:**
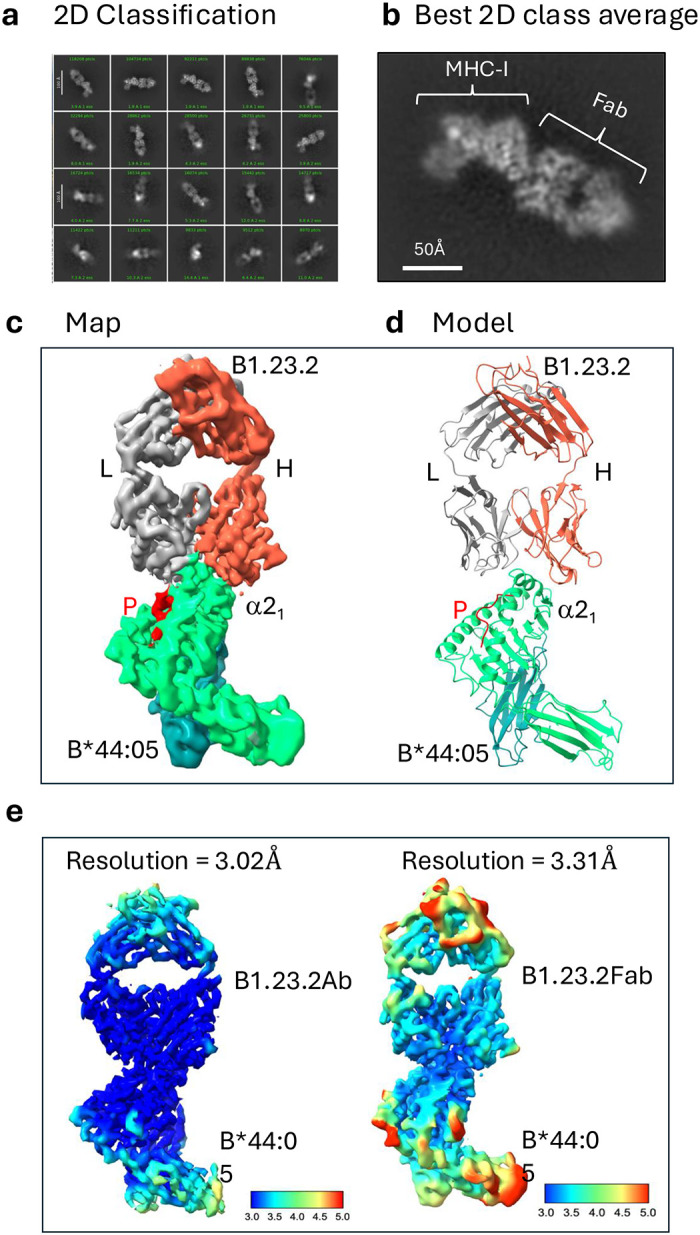
cryoEM structure of Fab B1.23.2 + HLA-B44:05. **a.** 2D classification after several runs of particle picking. **b.** The best 2D class clearly shows domains of Fab and MHC-I. **c.** The final refined map at 3.31 Å resolution, colored by domain, with red indicating the peptide. **d.** The refined model (PDB ID: 9D74) is compared with the map, with the same color indicating each domain and peptide. **e.** Local resolution maps reveal the best resolution in the interface area. Left: complex of mAb B1.23.2 and HLA-B*44:05, map-resolution = 3.02Å. Right: complex of Fab B1.23.2 and HLA-B*44:05, map-resolution = 3.31Å. Blue is high resolution; red, low resolution. The color scale bar indicates the resolution distribution.

In parallel with the structure determination of the complex of the full B1.23.2 mAb/HLA-B*44:05 as presented above, we also collected cryo-EM data for a complex of the Fab of B1.23.2 bound to HLA-B*44:05 and solved the structure to a resolution of 3.31 Å (Extended Data Fig. 4). The models derived from the two (Fab/B44:05 vs. full mAb/B44:05) are essentially identical (see Discussion).

### X-ray structure of B1.23.2 Fab/HLA-B*44:05 complex

We also determined the X-ray crystal structure of the complex of B1.23.2 Fab and HLA-B*44:05 (PDB ID: 8TQ6) (Extended Data Fig.6a) to a resolution of 3.20 Å. Data collection and refinement statistics are given in Table 2. Electron density maps (2mFo-DFc, contoured at 1.5σ) for the CDR loops of H chain and L chain of B1.23.2 Fab interacting with α2_1_ helix are shown in Extended Data Fig.6b. Comparison of the X-ray crystal structure (8TQ6) with the two cryo-EM structures (PDB IDs: 9D73 and 9D74) shows very little difference in RMSD or BSA values, with RMSD values of approximately 1.5 Å for all atoms (Extended Data Fig.6c). The BSA values at the antibody/antigen interfaces for X-ray crystal structure are slightly smaller than for the cryo-EM structures. Although the X-ray and cryo-EM-determined structures depend on fundamentally distinct methodologies and resolution is evaluated by different standards (see Discussion), the quality of the maps was very similar, and the identification of the interface residues of B1.23.2 with HLA-B*44:05 was in complete agreement.

### Peptide variants at position 8 influence affinity of B1.23.2 for HLA-B*44:05

**Extended Data Fig. 5 |:**
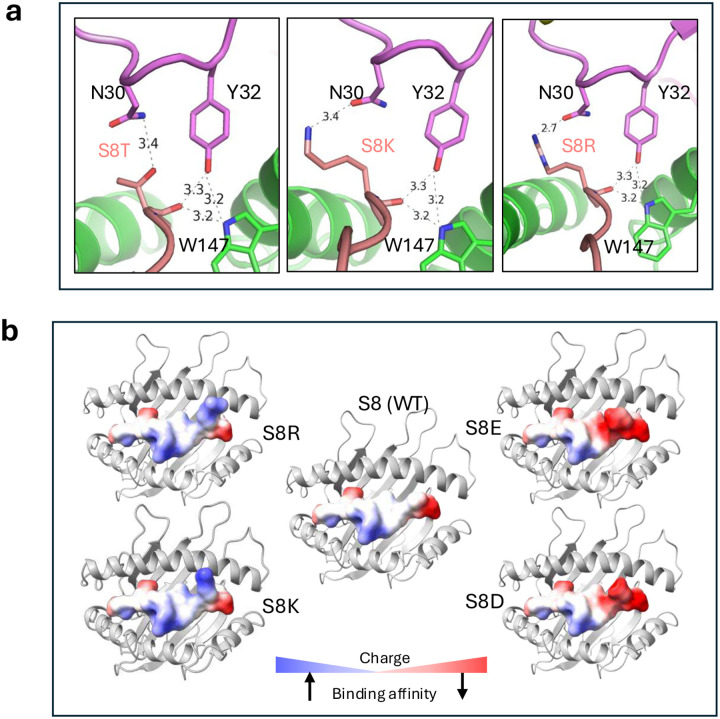
Peptide variants at position 8 influence affinity of B1.23.2 for HLA-B*44:05. **a.** Energy-minimized models of peptide variants S8T, S8K and S8R show the formation of the hydrogen bonds with the sidechain of N30 of B1.23.2. **b.** Electrostatic surface charge for S8K, S8R, S8E, and S8D is shown, illustrating that greater positive charge increases the binding affinity, while negative charge decreases the binding affinity, as indicated.

**Fig. 3 |:**
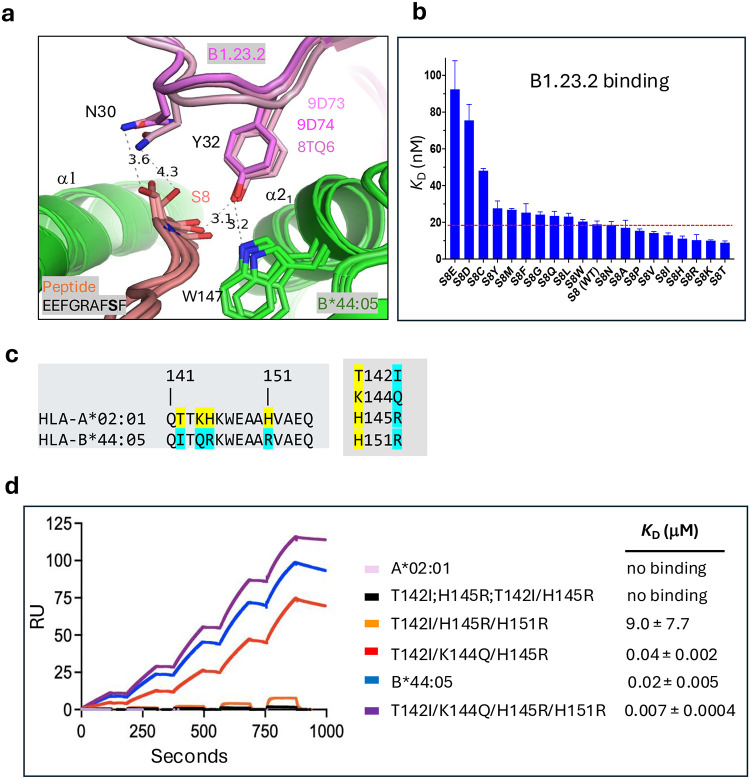
Peptide variants at position 8 and polymorphic residues of the α2 domain influence the affinity of B1.23.2 for HLA-B*44:05 HLA-A*02:04. **a.** Superposition of two cryo-EM structures (9D73 and 9D74) and the X-ray structure (8TQ6) reveals that Y32 of B1.23.2 L chain consistently contacts the carbonyl O of S8 of the peptide and recognizes W147 of HLA-B*44:05, forming a double hydrogen-bonded tripartite interaction. N30 of B1.23.2 L chain has a long-distance contact (3.6–4.3 Å) with the side chain of S8 of the peptide. **b.** The binding affinities (*K*_D_ (nM)) of B1.23.2 with 19 substitutions at S8 of the peptide of EEFGRAFSF to HLA-B*44:05. Value of the *K*_D_ for the complex containing the wild-type peptide is indicated. **c.** Sequence alignment on α2_1_ helix of HLA-B*44:05 and -A*02:01, colors highlight differences. **d.** The binding data of HLA-A*02:01, the indicated α2_1_ mutants, and HLA-B*44:05 to B1.23.2. On the right panel, the binding affinity, *K*_D_ (μM) is shown for each parental and mutant. Binding of α2_1_ mutants of HLA-A2 to B1.23.2 was determined by SPR.

Analysis of the interface between B1.23.2 and HLA-B*44:05 revealed direct contacts between Y32 of CDRL1 of B1.23.2 and the carbonyl O at peptide position 8 (S) (Fig. 3a). B1.23.2 Y32 also forms an H-bond to HLA-B*44:05 W147, a highly conserved HLA residue critical to orienting the penultimate peptide residue in almost all known MHC-I structures. Light chain CDRL1 residue N30 also has a long distance contact to the peptide S8 side chain. To evaluate the possibility that peptide variants at position 8 might influence the binding affinity of B1.23.2 to HLA-B*44:05, we refolded HLA-B*44:05 with each of 19 peptides with position 8 substitutions and evaluated their binding to B1.23.2 (Fig. 3b). The different complexes bound B1.23.2 with a spectrum of *K*_D_ values ranging from ~8 nM (for the T substitution) to about 90 nM (for E) – 10-fold weaker. Complexes prepared with acidic peptide amino acids at position 8 (E, D, and C) were weaker binders to B1.23.2, and T or substitutions with basic amino acids (K, R, and H) were slightly stronger (Fig. 3b). To understand the structural basis of these relatively small differences in affinity, we generated energy-minimized models of the structures with the substituted peptides (see Methods). Substitutions of the side chain at position 8 showed variation in the interaction with N30 of the B1.23.2 V_L_ CDRL1 (Extended Data Fig. 5a). Specifically, with T substitution, the position 8/N30 H-bond is slightly shorter, that with K is about the same, and R is drawn even closer to N30. The general surface charge difference around peptide residue 8 is evident in electrostatic surface calculations (Extended Data Fig. 5b).

### Mutations of epitopic residues on α2_1_ of A*02:01 -- experiment and computation

Knowing that the main epitopic residues of HLA-B*44:05 are those of the α2_1_ helix, and that HLA-A*02:01 and closely related allelomorphs (-A*02:03, -A*02:06, -A*68:01, -A*68:02, and -A*69:01) failed to bind B1.23.2 (Extended Data Fig. 1b), we transplanted HLA-B*44:05 residues onto the HLA-A*02:01 α2_1_ helix and tested binding to B1.23.2 (Fig. 3c). Four residues of HLA-A*02:01, T142, K144, H145, and H151 are of particular interest (Fig. 3c), so several single and multiple mutants were generated. The binding affinities of pMHC complexes containing these substitutions are shown in Fig. 3d. Single HLA-A*02:01 mutants T142I and H145R as well as the double T142I/H145R mutant did not show any binding. The triple mutations (T142I/H145R/H151R) and (T142I/K144Q/H145R) improved the binding affinities to *K*_D_ = 9.0 μM and *K*_D_ = 0.04 μM, respectively. The quadruple mutant (T142I/K144Q/H145R/H151R) bound even better than the HLA-B*44:05 molecule (*K*_D_ = 0.007 μM). These results indicate that HLA-A*02:01 can gain B1.23.2 reactivity upon localized substitutions in the epitopic region of HLA-A*02:01 consistent with the structural data.

We also performed molecular dynamics simulations (see Methods) to evaluate the contributions of the α2 domain differences between HLA-B*44:05 and HLA-A*02:01 to B1.23.2 recognition. Starting with a model of the V_H_ V_L_ domains of B1.23.2 bound to a fragment of HLA-B*44:05, we analyzed individual substitutions (HLA-B*44:05 to -A*02:01) I142T, Q144K, R145H, and R151H as well as the quadruple mutant (Extended Data Fig. 8a,b). The backbone root-mean-square-deviation (RMSD) trajectories and corresponding probability distributions were calculated for the α2_1_ helix and flanking residues of HLA-B*44:05 and the indicated mutants (Extended Data Fig. 8c). HLA-B*44:05 displayed a narrow RMSD distribution of 2.96 ± 0.64 Å (Extended Data Fig. 8d) and limited fluctuation (RMSF) (Extended Data Fig. 8e), indicative of a relatively stable structure throughout the simulation. In contrast, the mutants, especially Q144K and the quadruple mutant, showed broader and larger RMSD distributions (Extended Data Fig. 8c,d) reflecting increased conformational instability. In addition, the residue-wide RMSF of the V_H_ and V_L_ residues reveal greater flexibility due to mutation of the HLA epitope as compared with the complex with HLA-B*44:05 (Extended Data Fig. 8f,g). Thus, the MD simulations are consistent with the experimental binding characteristics of HLA-A*02:01 substitution mutants (Fig. 3d).

### Structural comparison reveals competition between anti-MHC-I mAb and inhibitory cell ligands

**Fig. 4 |:**
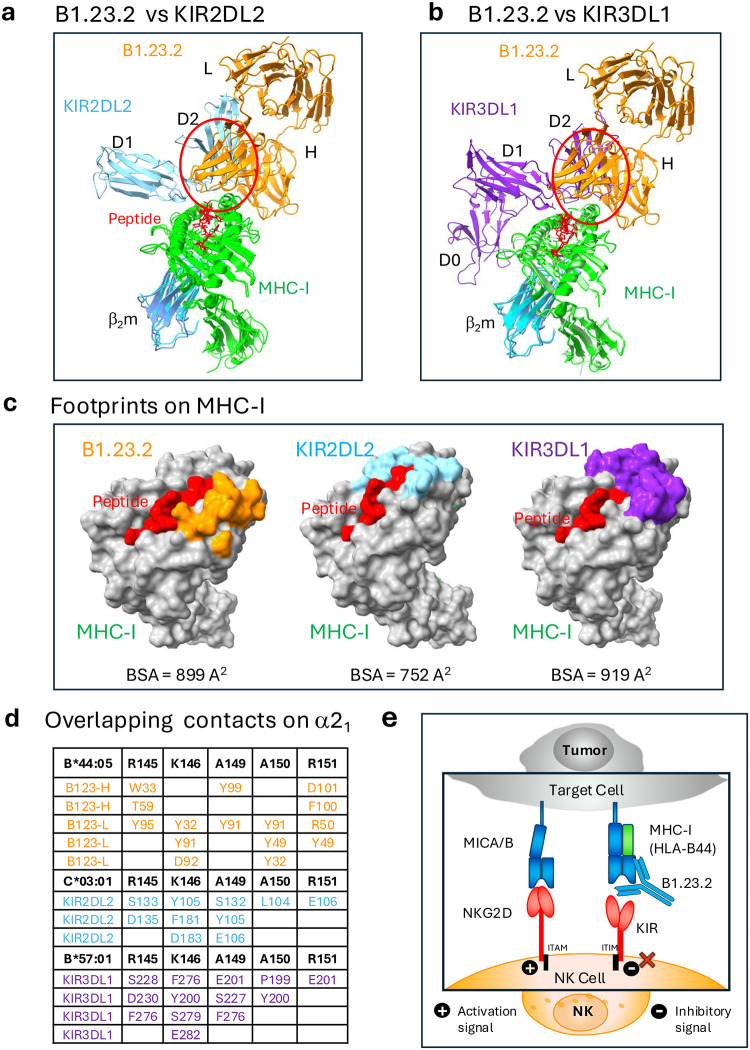
B1.23.2 mAb contacts on HLA overlap with those of KIR. **a.** Superposition of KIR2DL2+HLA-C*03:01 (PDB ID: 1EFX) on B1.23.2+HLA-B*44:05 (PDB ID: 9D73) indicates the clash of the D2 domain of KIR2DL2 over V_L_ and V_H_ domains of B1.23.2. **b.** Superposed KIR3DL1+HLA-B*57:01 (PDB ID: 3VH8) on B1.23.2+HLA-B*44:05 (PDB ID: 9D73) indicates the clash of the D2 domain of KIR3DL1 over V_L_ and V_H_ domains of B1.23.2. **c.** Footprints from B1.23.2 (orange), KIR2DL2 (cyan), and KIR3DL1 (blue purple) on the surface of MHC-I. The Buried Surface Areas (BSA) are 899Å^2^, 752Å^2^, and 919Å^2^ for B1.23.2, KIR2DL2, and KIR3DL1, respectively. **d.** Alignment of overlapping contact residues on the α2_1_ helix of HLA from B1.23.2 and KIR’s (see Contact Tables in Extended Data Table 2). Amino acid residues of B1.23.2 (H and L), KIR2DL2, and KIR3DL1 that interact with the indicated HLA-B or -C residues are tabulated, revealing that the several HLA ligands compete for interaction with conserved HLA residues R145, K146, A149, A150 and R151. **e.** A mechanistic model illustrating how anti-MHC mAb (B1.23.2) may compete with KIRs. When B1.23.2 binds to HLA-B*44:05, the interactions between KIRs and HLA are blocked, which may cancel the inhibitory signal, thus allowing the activation signal to dominate.

To gain further insight into the potential value of B1.23.2 to block HLA/KIR interactions, we examined the site of interaction of B1.23.2 in comparison with structurally determined footprints of KIR inhibitory receptors on MHC-I. Although several KIR/HLA structures have been determined, we selected two for detailed comparison. We superposed the HLA-B*44:05 heavy chain of the B1.23.2/HLA-B*44:05 complex onto the HLA-C*03:04 heavy chain of the KIR2DL2/HLA-C*03:04 complex (PDB-ID: 1EFX)^40^ (Fig. 4a) and onto HLA-B*57:01 of the KIR3DL1/HLA-B*B57:01 (PDB-ID: 3VH8) complex (Fig. 4b)^39^. Remarkably, the V_L_ domain of B1.23.2 completely overlaps with the D2 domain of either KIR2DL2 or KIR3DL1. The footprints of B1.23.2, KIR2DL2, and KIR3DL1 on the MHC-I surfaces are illustrated in Fig. 4c. The conserved KIR residues (S133/D135/E106-for KIR2D or S228/D230/E201-for KIR3D)^39,40,46^ of the D2 domain that interact with HLA R145, K146, and R151 are substituted by Y32/Y91/D92/Y95 of the L chain of B1.23.2 in the Ab complex (Fig. 4d). The contacts of B1.23.2, KIR2DL2 and KIR3DL1 overlap on five major residues: R145, K146, A149, A150 and R151 of the α2_1_ helix (Fig. 4d, Extended Data Table 2). Importantly, the binding affinity of B1.23.2 (*K*_D_ = 0.02 μM) is much higher than that of KIRs (*K*_D_ from 9.5 to 17 μM) for HLA (Extended Data Fig.1a). Since B1.23.2 sterically competes for the same conserved site on the HLA α2_1_ helix that is bound by KIR2DL2 and KIR3DL, it may result in blocking the inhibitory signal given by these receptors to the NK cell. These structural comparisons explain the observed competition of this mAb with KIR binding^13,32,33^. Unlike other mAbs that block target/NK cell interactions by binding of the mAb directly to the NK cell receptor^47–52^, B1.23.2 blocks by binding to the inhibitory ligand (the HLA molecule) itself (Extended Data Fig. 7c). Thus, a single antibody with great cross reactivity for most HLA molecules has the potential to reverse the KIR2DL or KIR3DL-mediated suppression of NK activity. This effect may be similar to that observed for pan anti-MHC-I mAbs, such as M1/42 in the mouse^11,53^, or W6/32 and DX17 in the human^13^ that block interactions of other inhibitory receptors on NK and myeloid cells. Therefore, we propose a simplified mechanism by which anti-HLA mAb may block the inhibitory receptors of NK cells, as shown in Fig. 4e. When B1.23.2 binds to HLA, the interactions between KIRs and HLA are impeded, which may result in canceling the inhibitory signal, thus enhancing the activation signal to suppress tumor growth.

### B1.23.2 blocks KIRs, unleashes NK cell activation, and suppresses tumor growth

**Fig. 5 |:**
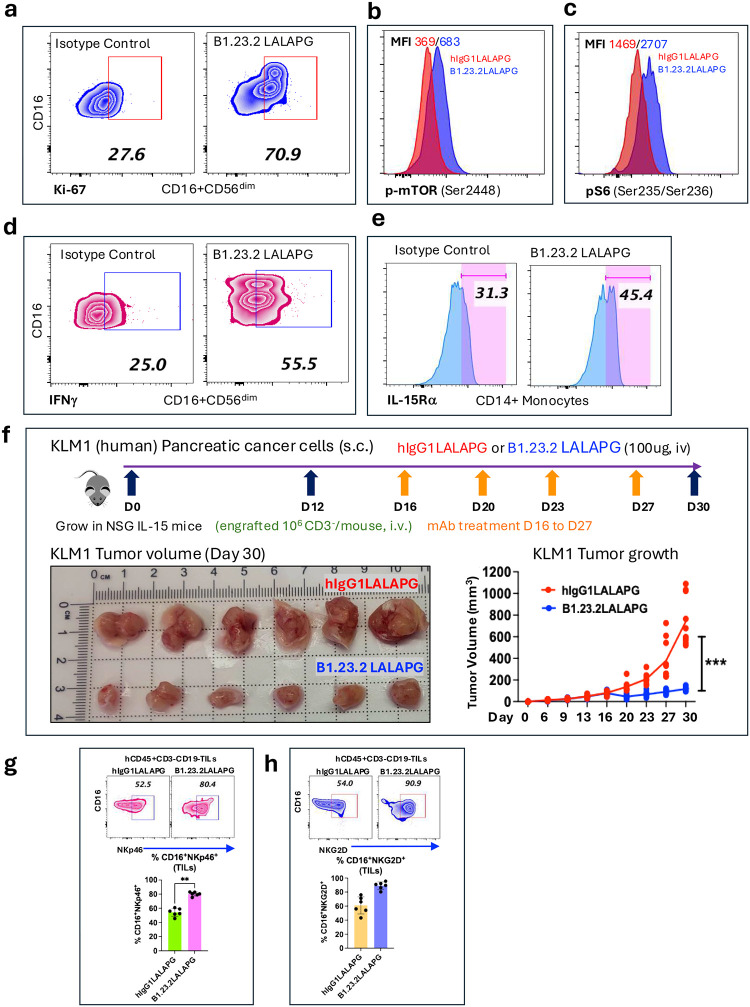
B1.23.2 treatment stimulates NK cell proliferation and augments anti-tumor immunity. **a.** Human PBMC cultured with control or B1.23.2 LALAPG mAb for 72 hr were analyzed for Ki-67 on CD16^+^CD56^dim^ NK cells. **b,c.** p-mTOR or pS6 staining of CD16^+^CD56^dim^ NK cells is shown, indicating MFI values. **d.** Intracellular IFNg staining on CD16^+^CD56^dim^ NK cells after 72 hr stimulation. **e.** IL-15Rα on CD14^+^ monocytes after 72 hr stimulation (% positive indicated). **f.** B1.23.2 LALAPG treatment controls human tumor (KLM-1) growth in NSG-IL15 mice. Treatment scheme illustrated (above) and day 30 tumor volume sizes are shown (upper row shows control hIgG1 LALAPG, lower row is B1.23.2 LALAPG (n=6)). MAb treatment started at Day 16. **g.** NKp46 and **h.** NKG2D expression on CD16+ TIL harvested on day 30. (statistics reflect nonparametric, Mann-Whitney t-test, using Graphpad Prism).

The structural mapping of the B1.23.2 binding site suggested that this mAb, by functionally blocking KIR2DL or KIR3DL interactions, would lead to NK cell activation. For these experiments we used the LALAPG-engineered B1.23.2, which fails to interact with any Fc receptors to avoid additional complicating factors. As shown in Fig. 5a, coculture of human PBMC with B.1.23.2 LALAPG leads to increased staining with Ki67 (26.7 to 70.9 %)(Fig. 5a), an indication of cell proliferation, increased mTOR and pS6 expression in NK cells (Fig. 5b,c), and enhanced levels of IFNγ (25.5 to 55.5 %) (Fig. 5d). In addition, CD14^+^ monocytes were activated to modestly increase their production of IL15Rα (Fig. 5e). To evaluate the potential of B1.23.2 in anti-tumor immunity, NSG-IL15 mice were given the KLM-1 human pancreatic tumor, then engrafted with human CD3^−^ cells, and monitored for tumor growth (Fig. 5f). Compared with isotype control treated animals, those that received B1.23.2 LALAPG showed significant reduction of tumor volume out to 30 days. Phenotypic analysis of the tumor infiltrating lymphocytes (TILs) from these tumors showed increased levels of activating natural cytotoxicity receptors, NKp46 (Fig. 5g) and NKG2D (Fig. 5h). Thus, B1.23.2, apparently by blocking interactions of NK inhibitory receptors on NK cells, results in tumor control in a humanized mouse model.

## DISCUSSION

Here we describe two cryo-EM structures and the X-ray structure of a highly cross-reactive anti-HLA mAb, B1.23.2, in complex with HLA-B*44:05. These provide a clear view of the contacts and explain how this Ab competes for KIR2 and KIR3 binding to HLA molecules. In addition to defining the HLA epitope, our analysis confirms the small influence of the HLA-bound peptide (position 8) to B1.23.2 binding, and explains the lack of specificity for a limited number of HLA-A*02:01 related allelomorphs. Study of several HLA-A*02:01 site-directed variants that gain binding activity not only confirms the identification of the epitopic region but also may provide insight for expanding the reactivty of B1.23.2 by selective mutagenesis. It is important to emphasize that B1.23.2 exhibits broad anti-HLA-A, -B, and -C reactivity but is distinct from several TCR mimic (TCRm) antibodies that exhibit restricted peptide and MHC recognition. Similar to W6/32 and DX17, pan anti-HLA antibodies that inhibit LILR interactions^13^, B1.23.2 has broad reactivity but for KIR2 and KIR3 binding sites.

### Comparison of B1.23.2 mAb/HLA-B*44:05 and B1.23.2 Fab/HLA-B*44:05 structures

X-ray crystal structures of relatively few full-length IgG antibodies with natural hinge regions have been reported (PDB IDs: 1IGT, 1IGY, 1HZH, 5DK3, and 6GFE)^54–58^. Two other complete structures were obtained of molecules with hinge deletions (Ab DOB^59^ and PDB ID: 1MCO^60^). Although cryo-EM offers to provide structural information of larger complexes than crystallography, few complete antibody complexes have been reported by this method. Most cryo-EM structures for antibodies appear as Fab despite efforts to use the full-length form of the antibody. Recently, cryo-EM structures of IgM Fc-pentamers (PDB ID: 6KXS, and 8BPE^61,62^), and a full-length IgM pentamer (PDB ID: 8ADY^63^) have been described. In negative stain images, IgG showed multiple forms of full-length antibodies with variation of the hinge anglebetween Fc and Fab and different elbow and rotation angles between Fabs^58^. The challenge presented by full-length antibodies for cryo-EM structure determination is the heterogeneity of such molecules due to the flexibility of the hinge joining the Fab and Fc domains. We did not directly observe the full length mAb B1.23.2 in complex with B*44:05 within a single reconstruction, but we were able to observe the Fab and Fc domains separately, from which we constructed a plausible full length model of the mAb-HLA complex (Extended Data Fig.4e). The full-length images were obtained with a recombinant B1.23.2 consisting of the native murine Fab region spliced to human Fc. Whether this unique construction contributed to our ability to visualize overlapping density from Fab and Fc images is not known.

Comparing the two cryo-EM structures of the B1.23.2 Ab/HLA-B*44:05 (9D73) and the B1.23.2 Fab/HLA-B*44:05 (9D74), we found that the cryo-EM map resolution for the B1.23.2 Ab/HLA-B*44:05 at the Fab-HLA interface is better (3.0Å) than that of B1.23.2 Fab/HLA-B*44:05 (3.3Å) (Extended Data Fig. 4e). This suggests that in some cases, using a full-length antibody for cryo-EM structure determination may prove more favorable than using the Fab alone. In general, using the Fab rather than the whole mAb for the crystallization of complexes is more successful.

In a comparison of cryo-EM and X-ray crystal structures, we noticed that the B-factors of the cryo-EM structure are larger than those of the crystal. This reflects the inherent flexibility and dynamic nature of biomolecules in the solution state, frozen in ice that may result in greater background noise, while X-ray crystallographic structures, due to the solid state of the crystal lattice, tend to have relatively lower B-factors. In addition, resolution in the crystal structure is estimated from the X-ray data (obtained in reciprocal space) that represent an overall value for the whole electron density map, and the B-factor describes the uncertainty or mobility around each atomic center. The map resolution evaluated by FSC in a cryo-EM structure is visualized in the density map (real space), but it represents a likely maximum resolution of the map, and the B-factor in the cryo-EM structure describes the density distribution around the atomic center. Unlike the Free-*R* value^64^ that is used in validation of X-ray crystal structures, the Q-score^65^ is generally employed to validate the model fit to the map in a cryo-EM structure. We observed that the map resolution at the interface of the complex for a cryoEM structure is higher than that for the surface (Extended Data Fig. 4e). Binding forces (interactions between the antibody and MHC-I) may partially stabilize the flexible CDR loops at the interface.

### Correlation and competition between TCR, inhibitory receptors, and anti-MHC-I mAb

One application of mAb B1.23.2 of therapeutic value is to exploit its potential for blocking inhibitory receptor/HLA interactions. Since the site of B1.23.2 overlaps extensively with the interface exploited by KIR2 and KIR3 receptors, it is effective in activating NK cell populations. In general, αβTCR, although as a group they describe a range of α1, α2, and peptide footprints, by and large cover the same region as seen by B1.23.2^9^. By contrast, γdTCR reveal a wider variety of interaction sites on pMHC in part reflecting their ability to bind non-classical MHC-I molecules such as CD1 or MR1^66^. It is noteworthy that B1.23.2 fails to bind the most common HLA allelomorph, HLA-A*02:01, and this characteristic may prove valuable as an adjunct to HLA-A*02:01-restricted CAR-T cell therapy by augmenting NK cell responses. In Extended Data Fig. 7a we illustrate the contact residues on pMHC-I seen by TCR, KIR, and B1.23.2, respectively, and how they overlap. KIR binds many of the same MHC-I residues as do TCR and also interacts with at least two peptide residues. B1.23.2 overlaps with KIR only on the α2_1_ helix and one peptide residue at position 8. Five residues on the α2_1_ helix would be expected to interact with some αβTCR^9,67^, but not all γdTCR^66^. Extended Data Fig.7b illustrates a mechanistic model for the competitive relations of the anti-MHC-I mAb and the inhibitory receptor of NK cell, as a mechanism distinct from the effects of other checkpoint inhibitors (anti-PD1 or anti-PDL1) (Fig. 7c).

Despite exceptional progress in the treatment of a host of malignancies using antibodies directed against tumor-associated and neoantigens^68^, the striking effects of antibodies that function as checkpoint inhibitors^69^, and the rapid development of strategies to engineer bespoke reagents such as CAR-T cells^70^, there remains room for approaches that may harness NK and myeloid cell activation to augment cancer treatments^71^. Several groups have developed anti-inhibitory receptor mAb to block HLA/KIR or HLA/NKG2A interactions^32,49,50,72^. We have previously shown that the pan anti-MHC-mAbs, M1/42 in the mouse, and W6/32 and DX17 in the human, that block engagement of tumor cells by the Ly49 or LILR inhibitory receptors on NK or myeloid cells, result in strong anti-tumor responses^11–13^. Here we show that another widely reactive mAb, B1.23.2, binds almost all HLA-A, -B, and -C molecules at a site distinct from the W6/32, DX17 site, and which blocks KIR2 and KIR3 interactions with HLA, and has the potential to augment NK cell activity. Further studies of this and similar mAbs offer a novel avenue to complement current therapies. Here, from a structural viewpoint, we describe a highly cross-reactive anti-HLA class I mAb (B1.23.2) that can block inhibitory KIR/HLA interactions and has the potential to add to a multipronged approach to tumor treatment. It is unlikely that any single antibody can result in effective activation of innate immune cells by blocking inhibitory receptors throughout the full course of therapy, but different antibodies directed against different inhibitory ligands and checkpoint inhibitors may contribute to progress in the course of treatment. The particular value of antibodies like B1.23.2 is that at while blocking KIR/HLA interactions, they would spare the interaction of clonotypic TCR recognizing HLA-A*02:01-peptide complexes. Further studies of this and similar mAbs offer a novel avenue to complement current therapies.

## METHODS

### Recombinant proteins

The B1.23.2 hybridoma (mouse IgG2a)^30^ was a kind gift of Dr. Bernard Lafont. RNA was extracted from 10^7^ cells grown in DMEM (Lonza) supplemented with 10% fetal calf serum, glutamine, non-essential amino acids, HEPES, and 50 μg/ml gentamicin, using a total RNA extraction kit (New England Biolabs) following manufacturer’s instructions. cDNA was prepared from 5 μg total RNA with the One-Taq RT PCR kit (New England Biolabs) following manufacturer’s protocol. A panel of mouse immunoglobulin V-gene PCR primers^73^ was used to amplify the expressed H and L chain V genes by RT-PCR followed by DNA sequencing. DNA encoding the H chain V sequence fused to the C_H1_ domain of human IgG1 was synthesized by Genscript (Genscript USA) and cloned by InFusion cloning (Takara) into a pcDNA3.1(+) vector encoding the human IL-10 signal sequence and the C_H2_ and C_H3_ domains of human IgG1. To prevent interaction with mouse and human Fc receptors three mutations, L234A, L235A, and P329G^74^ were introduced in the Fc region of B1.23.2. Similarly, DNA encoding the L chain V sequence was fused to mouse Ck and cloned into pcDNA3.1(+) downstream of the human IL-10 signal sequence. H and L chain plasmids were transfected into exponentially growing HEK293S cells using ExpiFectamine^™^ and enhancers (GIBCO) following manufacturer’s protocol. Seven days after transfection, secreted antibody was affinity purified on Protein A Sepharose (Cytiva) followed by size exclusion chromatography on Superdex 200 (Cytiva) in 25 mM TRIS pH 8, 150 mM NaCl. This recombinant full-length antibody is referred to as “mAb B1.23.2” throughout the paper.

To prepare Fab, 20 mg of the mouse antibody (purified from B1.23.2 hybridoma supernatant) was digested with immobilized papain (Thermo Scientific) for 4 hours at 37 °C in phosphate buffered saline (PBS) containing 20 mM cysteine and 2 mM EDTA. Following dialysis overnight against PBS, Fc fragments and undigested antibody were removed by Protein A Sepharose (Cytiva) chromatography and the Fab preparation was further purified by size exclusion chromatography on Superdex 200 (Cytiva).

Bacterial expression, refolding, and purification of the luminal domains of HLA-B*44:05 complexed with human β_2_m and the nonamer peptide, EEFGRAFSF representing residues 46–54 of the human HLA-DPA1*02:01, were performed as described previously^75^. Similar expression, refolding and purification for each of 19 peptide variants at P8 of the peptide was accomplished.

### Surface Plasmon Resonance (SPR)

SPR experiments were carried out in a BiaCore T200 (Cytiva, Uppsala, Sweden) at 25 °C in 10 mM TRIS pH 7.4, 150 mM NaCl, 3 mM EDTA, and 0.05% Surfactant P20. B1.23.2 antibody was covalently coupled to a CM5 chip to 600 RU via amine coupling chemistry with EDC/NHS. Using the single cycle kinetics method, serial 2-fold dilutions of MHC-I ranging from 500 nM to 31.2 nM were sequentially injected over the antibody surface at a flow rate of 20 μL/min. Each injection was for 120 seconds followed by a 120 second dissociation phase before the next MHC-I concentration was injected. Regeneration was carried out at the end of the final cycle with a 15 second injection of 0.1M glycine/0.15M NaCl pH 2.3 at 30 μL/min. Binding experiments were repeated three times. Sensorgrams were globally fit to a 1:1 binding model with BiaCore T200 Evaluation Software 3.1 and plotted with Prism (GraphPad Software, San Diego, CA, USA).

### Screening of reactivity of B1.23.2 for binding to a panel of HLA-A, -B, -C molecules.

Single antigen beads (SABs) are fluorescently color-coded and coated with 97 different HLA allotypes loaded with peptides derived from Epstein-Barr virus (EBV)-transfected cell lines. Biotinylated mAbs B1.23.2 and W6/32 were tetramerized using Streptavidin–phycoerythrin (PE; Agilent Technologies Inc.) at a final concentration of 0.5 mg/mL. The resulting B1.23.2 and W6/32 tetramers were each diluted at a staining ratio of 1:50 and mixed with 4 μL of LABScreen SAB suspension (OneLambda Inc., CA, USA) to a final volume of 24 μL in a 96-well plate. Samples were then incubated with either B1.23.2 or W6/32 tetramer for 1 hour at RT with shaking at 550 rpm, washed four times with wash buffer (OneLambda Inc., CA, USA) to remove excess tetramers, and resuspended in phosphate-buffered saline (PBS; pH 7.2). The mean fluorescence intensity (MFI) of SABs upon incubation with B1.23.2 or W6/32 was measured by the Luminex 100 Liquid Array Analyzer System. The experiment was conducted in duplicate. The MFI ratio was then calculated by dividing the average MFI of B1.23.2 by the average MFI of W6/32. The results were analyzed and plotted in GraphPad Prism v10.

### Flow cytometry

For surface staining, HeLa cells (1 × 10^6^) that express individually tranfected HLA molecules, in staining buffer (PBS, 10% heat-inactivated FBS, and 0.05% sodium azide), were incubated with the following surface conjugate antibodies in 1:50 dilutions: anti-HLA-A2 (mAb BB7.2, BD Biosciences 568757), Anti-HLA-B (mAb YTH 76.3, BD Biosciences 567211), Anti-HLA-C (DT-9, BD Biosciences 566372), Anti-HLA-E (3D12, BD Biosciences 567418), Anti-HLA-G (87G, Biolegend 335912), B1.23.2 (ThermoFisher 17-5935-42) and DX17 (BD Biosciences 560169) in the presence of Fc block (BD biosciences). Cells were washed with FACS staining buffer and acquired by LSRFortessa (BD Biosciences) flow cytometer with FASCDiva software and analyzed by FlowJo (tree star version 10) software.

hPBMC from healthy donors were suspended in complete RPMI-1640 medium. For surface staining, the cells were initially blocked using FCX Trustain (Biolegend) for 5 minutes, followed by staining with the surface conjugate for 20 minutes in FACS staining medium (PBS, 10% heat-inactivated FBS, and 0.05% sodium azide). Intracellular staining was done by fixing and permeabilizing the surface-stained cells with eBioscience^™^ Foxp3 / Transcription Factor Staining Buffer Set (ThermoFisher Cat # 00-5523-00) for 15 minutes and then staining with Ki-67 (BD Bioscience: Cat No 571538), p-mTOR (ThermoFisher Cat # 25-9718-42) and pS6 (ThermoFisher Cat # 48-9007-42) antibodies. To perform intracellular IFNγ staining, PBMC in complete RPMI medium were stimulated with a cocktail of cell stimulation and protein transport inhibitors (Thermo Fisher Cat # 00-4975-03) containing PMA, ionomycin, Brefeldin A, and monensin for 3–4 hours at 37°C. Cells were washed, fixed, permeabilized, and stained with anti-IFNγ antibody (BD Biosciences Cat # 554701) overnight at 4°C. Finally, the cells were washed and analyzed using BD LSR Fortessa X-20 cell analyzer and BD FACSDiva Software version 8.0. Data further analyzed by Flowjo^™^ V10 (Treestar).

### Mice

NSG-IL-15 mice (NSG-IL15, stock no. 030890) were purchased from Jackson Laboratories and housed under specific pathogen-free conditions. All mice were sex and age matched and used between 10 and 13 weeks of age. All animal protocols used in this study were approved by the National Institute of Allergy and Infectious Diseases Animal Care and Use Committee (Protocol # LISB 15E).

### hPBMC culture with B1.23.2LALAPG mAb

Human peripheral blood mononuclear cells (hPBMC) were obtained from unidentified normal human blood bank donors. These studies are exempt from further ethical review by and Institutional Review Board and were performed according to guidelines of the National Institute of Allergy and Infectious Diseases. Cells were suspended in RPMI 1640 complete medium supplemented with 10% FBS, 2 mM L-glutamine, 1 mM sodium pyruvate, 1 mM HEPES, 0.1 mM nonessential amino acids, 50 μM 2-mercaptoethanol, and 100 U/ml penicillin and streptomycin. 10 mg of B1.23.2 LALAPG or hIgG1LALAPG was added to 0.5×10^6^ mononuclear cells in 500 μL medium in 48-well plates (Falcon) and incubated for 72 hours.

### Humanized mouse tumor model

KLM1 cancer cells were grown in DMEM medium supplemented with 10% heat-inactivated FBS, L-glutamine (2mM), sodium pyruvate (1mM), HEPES (10mM), non-essential amino acids (0.1mM), 2-mercaptoethanol (50μM), and penicillin and streptomycin (100U/ml). Sex- and age-matched hIL15 transgenic mice were injected subcutaneously (right flank region) with 1.5 × 10^5^ KLM1 cells suspended in 100 μL of sterile PBS. After 12 days of tumor cell engraftment, 10 × 10^6^ CD3^−^ hPBMC, isolated from healthy individuals by negative selection on magnetic beads (Miltenyi Biotec), were retro-orbitally injected into the tumor-bearing mice. hIgG1LALAPG and and B1.23.2LALAPG (100μg in 100μl PBS) were administered retro-orbitally (i.v.) twice a week for two weeks. Tumor size was measured in three dimensions regularly using digital calipers, and tumor volumes were calculated by multiplying length x width x depth. On day 30 post tumor implant, tumors were excised under sterile conditions, and TILs were prepared after mincing the tumor into very small pieces and further dissociated by Gentle MACS tissue dissociator. The dissociated tissue was passed through a 70mm strainer (BD Falcon), washed and then treated with ACK lysing buffer to lyse RBC. The single-cell suspension of TILs was washed with FACS staining buffer and purified by percoll density gradient centrifugation.

### Cryo-EM sample preparation and data collection

Freshly purified anti-human antibodies (full length “mAb B1.23.2 LALAPG” or Fab “Fab B1.23.2”) were incubated in a 1:1 molar ratio with bacterially expressed and refolded soluble peptide/HLA-B*44:05/hβ_2_m complexes prepared as described previously^75^. The complexes were purified by size exclusion chromatography (SEC) and used at a concentration of 0.7–1.4 mg/ml for sample preparation. Samples were applied onto holey-carbon cryo-EM grids (C-flat^™^ Holey Carbon Grid Gold 1.2 μm/1.3 μm space 300 mesh (Protochip, NC USA)), which had been glow discharged for 60 seconds, blotted for 3 seconds, and plunged into liquid ethane with a Vitrobot Mark4 (Thermo-Fisher) at 4 °C and 95% humidity. Cryo-EM data on the selected regions with ideal ice thickness were collected on a Titan Krios 300-keV microscope (NICE/NIH Cryo-EM consortium). Images were acquired automatically with SerialEM^76^ on a BioQuantum-K3 detector (Gatan) in super-resolution mode at 130x nominal magnification (0.83 Å/unbinned pixels) and a defocus range from −0.7 to −2.0 μm. An exposure time of 0.05s per frame was recorded, with a total exposure of about 54.2 electrons/Å^2^. Three raw data sets were collected: mAb B1.23.2+HLA-B*44:05 with 7,154 movies, and Fab B1.23.2+HLA-B*44:05 with 3,077 movies.

### Image processing, map resolution improvement, and model fitting

All image processing, 2D class, 3D reconstruction, and map refinements were performed using cryoSPARC v4.4.1^42,77,78^. Following “Patch Motion Correction,” “Patch CTF Estimation,” and “Curate Exposures,” outliers of defocus range, defective micrographs, and low-resolution estimation of the CTF fit (>6 Å) were discarded. The “Blob Picker” was initially used with a particle diameter of 128 Å for picking particles. The box size used for 2D classification and following was 256 pixels. The initial “Blob Picker” resulted in only a few 2D classes with suitable particles. Subsequently, we used these initial 2D classes as templates for “Template Picker” and, following standard protocols (“Ab-Initio Reconstruction” and “Non-uniform Refinement”) with several iterations, obtained a map resolution of about 4.0 Å for these antibody complexes.

### A protocol for map resolution improvement for small molecular weight complexes

We developed a protocol for map resolution improvement by using cryoSPARC^42^ as shown in Extended Data Fig. 2. A key parameter, “LowPass Filter” (LPF), for picking potent particles from the micrographs in the “Template Picker” procedure can be optimized (the default value is 20). LPF implies “signal/noise frequency”^77,78^. We used multiple “Template Picker” procedures with various LPF parameters (from 20 to 5) concurrently. Followed by the standard “extract particle” and “2D classification”, we may select some better 2D classes as the “template particles” for the next round of the multiple Template Picker procedure. An additional 2D classification was necessary to merge all selected particles and remove duplicates. The 3D classification could also remove some 3D groups with low-resolution particles. This protocol improves the map resolution, although it costs more computing time. We used multiple “Template Picker” with LPF (a,b) = (20,20); (15,15); (10,10); (5,5), where (a,b) is applied to (template, micrograph) respectively. The input particle templates were from previously refined particles. For the example of the mAb B1.23.2 + HLA-B*44:05 complex, we used concurrent Template pickers, iterating 5 times, that extracted 1,525,615 particles, selected 664,724 particles, and removed duplicates, leaving 516,068 particles for Ab-Initio construction. By eliminating poor conformations and optimizing the 3D classification, 359,876 particles were used for the final refined map (Fig.2d). The map resolution was improved to 3.02 Å (see Table 1). The same protocol was applied to the B1.23.2 Fab+HLA-B*44:05 data sets, and the final map resolution was improved to 3.31 Å (see Table 1), respectively.

### Crystallization, Data Collection, and Refinement

Crystallization conditions were identified by screening hanging drops at 18 °C. Crystals of Fab B1.23.2+HLA-B*44:05 were obtained under 16% PEG 3350, 0.04M Na citrate, 0.06M Bis-Tris pH 8.8. However, crystals of mAb B1.23.2+HLA-B*44:05 first appeared pin-like or needle-like and failed to provide usable diffraction data. We finally obtained relatively larger crystals by seeding with the reservoir well diluted with 20–30% water. Crystals were cryoprotected in mother liquor containing 10% ethylene glycol and flash frozen in liquid nitrogen. Diffraction data were collected (at wavelength 1.033 Å, in N_2_ stream at ~ 100 K) at Southeast Regional Collaborative Access Team (SER-CAT) beamline 22ID at the Advanced Photon Source, Argonne National Laboratory and processed with XDS^79^ to 3.2 Å resolution for Fab B1.23.2+HLA-B*44:05 (Table 2). The final data set for Fab B1.23.2+HLA-B*44:05 was merged from 3 data subsets with different omega angles for greater completeness and higher resolution. The structures were solved by molecular replacement with Phaser^80^ using H2-D^d^ from PDB 5WEU and HLA-B*44:05 from PDB 7TUC as search models. We used the DX17 Fab (PDB-ID: 8TQ5) model with CDR loops trimmed off as an initial Fab search model, then manually rebuilt the CDR loops according to electron density and amino acid sequence. These molecular replacement models were subjected to several rounds of refinement with Phenix^81^ interspersed with manual building in Coot^82^. All Fab sequences were established by PCR sequencing. R_work_/R_free_ (%) values for final refined models of Fab B1.23.2+HLA-B*44:05 are 24.2/27.5. Data collection and refinement statistics are summarized in Table 2. Ramachandran statistics for the final model of Fab B1.23.2+HLA-B*44:05 are 92.9, 6.2, and 0.9 for % favored, allowed, and outliers, respectively. Graphics figures were generated with PyMOL^83^ and ChimeraX^84^.

### Cryo-EM structure determination and refinement

We used the X-ray crystal structure model (PDB: 8TQ6) to dock and manually fit the cryo-EM maps of mAb B1.23.2 +HLA-B*44:05 and Fab B1.23.2+HLA-B*44:05, respectively. We used Real-Space Refinement in Phenix^85^, which includes rigid-body refinement. The MHC-I rigid-body domains consist of α1α2+peptide, α3, and β_2_m domains, and Fab consists of four rigid-body domains (V_L_, Cκ, V_H_, C_H1_). Simulated annealing (SA) at the initial step, local grid search, and ADP refinement were included. Secondary structure restraints were applied. The final refined model compared with the map densities has an overall CC (Correlation Coefficient) of 0.84/0.84/0.75 (mask/volume/peaks) for mAb B1.23.2+HLA-B*44:05, and 0.76/0.76/0.68 for Fab B1.23.2+HLA-B*44:05, respectively. We also calculated Q-score of individual residues^65^ for validation. Cryo-EM Data processing, refinement statistics, and model validation are listed in Table 1.

### Computational methodology for molecular dynamics (MD) simulation

To investigate the specific interactions and dynamic behavior of the α2_1_ helix of HLA-B*44:05 in complex with the antibody B1.23.2, we employed a restricted model that includes a fragment of the α2 domain (residues 127–158), along with the variable heavy (V_H_) and light (V_L_) chains of the B1.23.2. (Extended Figure 8). Classical MD simulations were performed using the cryo-EM structure of the anti-MHC-I monoclonal antibody B1.23.2 in complex with HLA-B*44:05 (PDB ID: 9D73). The initial coordinates were obtained from the PDB structure. Five HLA-B*44:05 point mutants, I142T, Q144K, R145H, R151H, and a quadruple mutant were generated using the mutagenesis tool in PyMOL by substituting residues of HLA-B*44:05 with the corresponding residues from HLA-A*02:01. Parameterization of all systems was carried out using the CHARMM-GUI interface with the CHARMM36 force field^86,87^. Each system was solvated using the TIP3P water model, ensuring a minimum buffer of 10 Å between solute and the box edge^88^. The periodic boundary condition box dimensions were set to 75 × 75 × 75 Å^3^, and sodium and chloride ions were added to neutralize the systems and mimic physiological ionic strength (pH 7.4). Each system contained approximately 38,913 water atoms. Simulations were performed using the NAMD 3.0.1 simulation package^89^. Long-range electrostatics were treated using the Particle Mesh Ewald (PME) method, and a cutoff of 10 Å was applied for van der Waals interactions^90^. Temperature and pressure were maintained at 300K and 1atm, respectively, using the Langevin thermostat and barostat. Initial energy minimization was conducted for 250,000 steps using the conjugate gradient algorithm to eliminate steric clashes and achieve a low-energy conformation. After energy minimization, the solvent equilibration was performed for 1ns at 300K at NVT ensemble. At each integration step, the velocities were reassigned from a new Maxwell distribution and the temperature was incremented by 0.0001K. To gradually relax the system, harmonic restraints were applied to protein atoms and reduced stepwise (99, 25, 1.0, 0.1, and 0.001 kcal/mol·Å^2^) over 1.25 ns. The SHAKE algorithm was used to constrain all bonds involving hydrogen atoms. A time step of 2fs was used for integration the equations of motion. Finally, production simulations were carried out under the NPT ensemble for 100 ns for each system. All trajectory analyses and molecular visualizations were performed using Visual Molecular Dynamics (VMD 1.9.1)^91^.

## Extended Data

**Extended Data Fig. 6 |:**
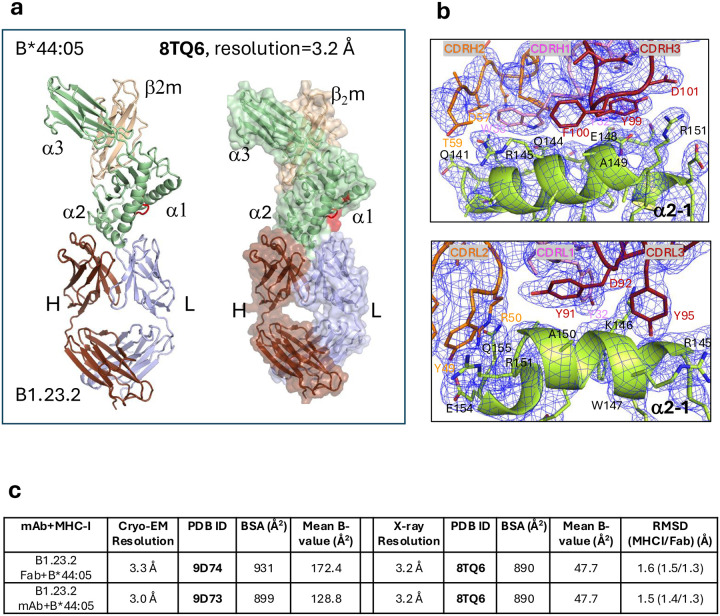
X-ray structure and comparison with Cryo-EM structures. **a.** X-ray crystal structure of Fab of B1.23.2 in complex with HLA-B*44:05 (PDB-ID: 8TQ6). **b.** Electron density maps of X-ray crystal structure of Fab of B1.23.2 in complex with HLA-B*44:05 (PDB ID: 8TQ6), [2mFo-DFc map contoured at 2.5 σ]: top panel shows that CDR loops of the H chain interact with the α2_1_ helix, and lower panel shows CDR loops of the L chain interacting with the α2_1_ helix. **c.** The differences in RMSD and BSA between the X-ray crystal and the two cryo-EM structures.

**Extended Data Fig.7 |:**
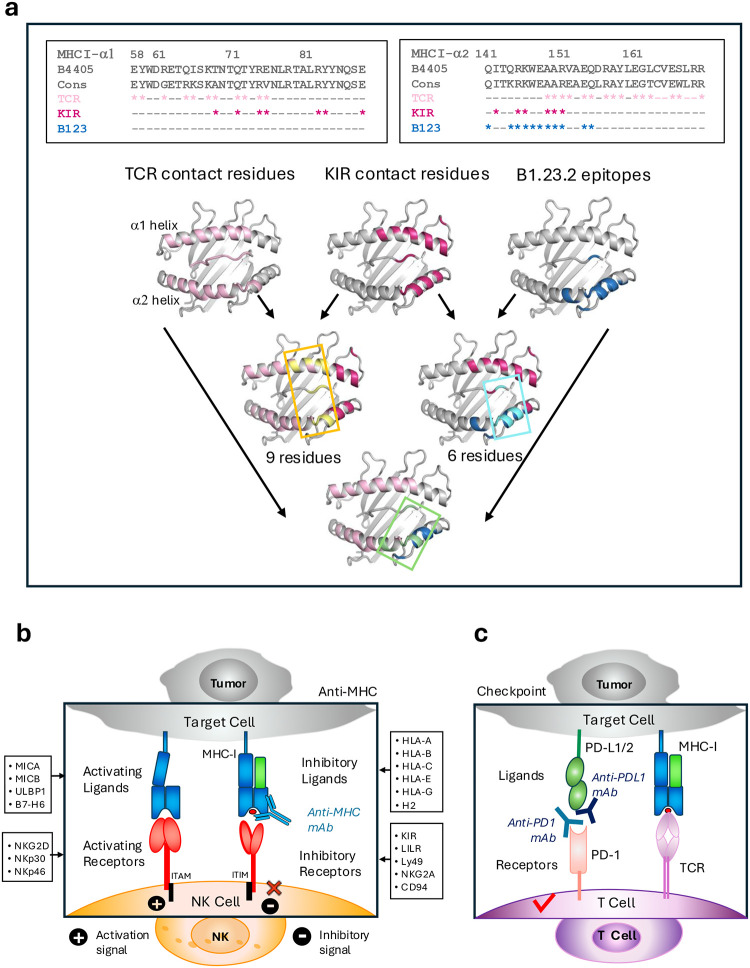
Overlapping contact residues on MHC-I by TCR or KIR with the epitopes of B1.23.2, and a model of the mechanism of anti-MHC-I mAb competition **a.** In the top panel, alignment of the epitopes (contact residues or binding sites) to HLA-B*44:05 and the consensus sequences. Stars indicate contact; dash indicates none, pink indicates TCR, warm pink indicates KIR, and blue indicates B1.23.2, yellow indicates the overlap between TCR and KIR, cyan means the overlap between KIR and B1.23.2, and pale green represents the overlap between TCR and B1.23.2. KIR contacts two peptide residues at P7 and P8, B1.23.2 contacts only one residue at P8, while TCR contacts all eight peptide residues from P1 to P8. TCR contact residues are from the majority of αβTCR (representative PDB IDs: 10GA, 1AO7, 1BD2, 1LP9, 2BNQ, 3HG1, 3GSN,^67^. KIR contact residues are from KIR2DL2 (PDB ID: 1EFX) and KIR3DL1 (PDB ID: 3VH8). **b.** A generalized mechanistic model of inhibitory receptor and anti-MHC-I mAb competition for binding to MHC-I. When the anti-MHC mAb binds to MHC-I, it blocks the interaction between MHC-I and the inhibitory receptors (KIRs/LILRs/Ly49s), which may lead to canceling the inhibitory signals, and enhancing the activation signals, resulting in the downstream killing and tumor suppression. **c**. An illustration of anti-PD1/PD-L1 mAb in the checkpoint pathway. When the anti-PD-1/PD-L1 mAb blocks the interaction between PD-1 and PD-L1, it leads to the activation of T cell activity, leading to tumor cell death^92^.

**Extended Data Fig.8 |:**
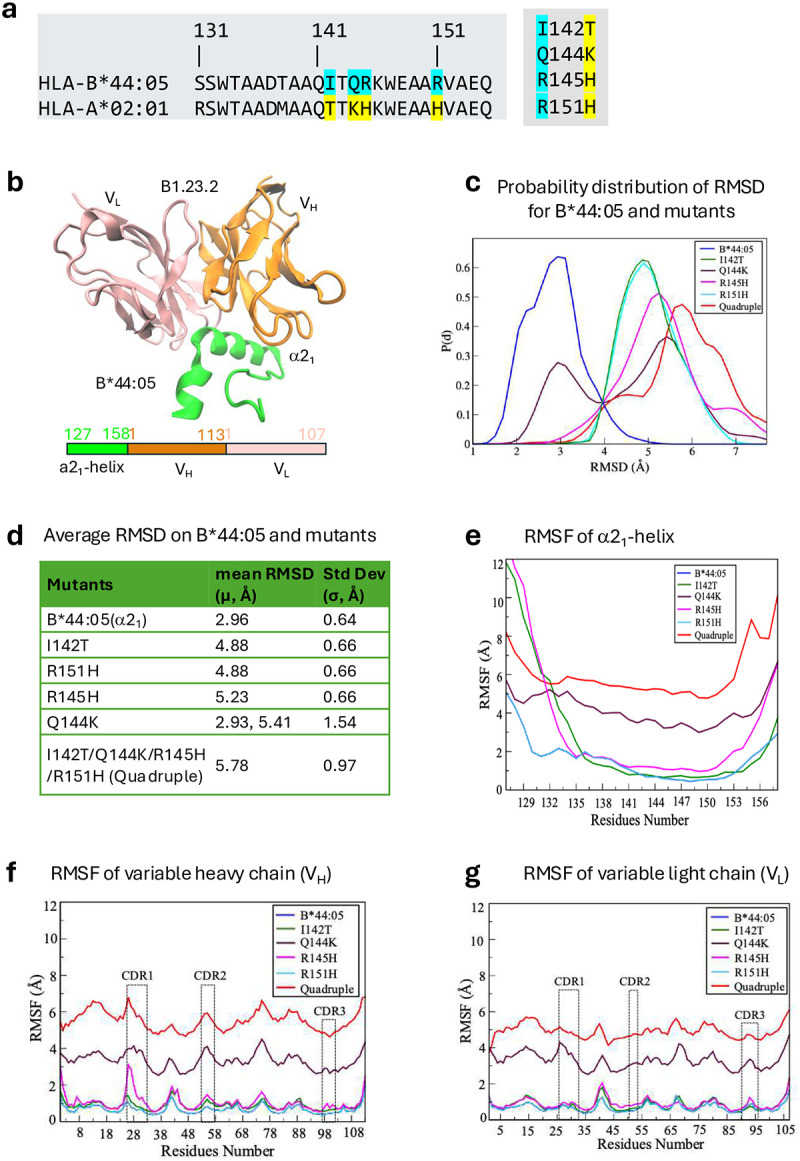
Molecular Dynamics simulations with mutations of four epitope residues on α2_1_ helix of HLA-B*44:05 in binding to B1.23.2. **a.** Sequence of the α2_1_ helix (residues 131–155) of HLA-B*44:05 aligned with HLA-A*02:01. Colors (cyan to yellow) indicate positions where substitutions were introduced. **b.** Ribbon view of α2_1_ fragment of HLA-B*44:05 (residues 127–158), V_H_ (residues 1–113, orange), and V_L_ (residues 1–107, pink) of B1.23.2 mAb. **c.** Conformational probability distribution (P(d)) of RMSD values for HLA-B*44:05 and its mutants. HLA-B*44:05 shows a narrow and sharply peaked distribution, indicating stable structural conformation throughout the simulation. In contrast, the other mutants, especially Q144K and Quadruple, exhibit broader distributions or shifted peaks, reflecting increased conformational flexibility and possible structural deviation from native structure. **d.** The mean RMSD and SD values for HLA-B*44:05 and mutations. The large average RMSD indicates the dissociation of the mutants, particularly of the Quadruple that agreed with the mutagenesis experiments in Fig. 3d. **e.** RMSF analysis of the α2_1_ fragment of HLA-B*44:05 and its mutants. **f.** RMSF of the variable heavy (V_H_) chain of B1.23.2 in complex with HLA-B44:05 and its mutants. **g.** RMSF of the variable light (V_L_) chain of B1.23.2 in complex with HLA-B44:05 and its mutants. RMSF values represent the positional flexibility of each residue over the course of molecular dynamics simulations. The Q144K and quadruple mutants exhibit markedly increased fluctuations in the α2_1_ fragment, indicating local destabilization or enhanced flexibility. In the V_H_ chain, regions corresponding to complementarity-determining regions (CDRs), CDR1, CDR2, and CDR3 (dashed lines), show significantly higher RMSF values for the Q144K and quadruple variant, reflecting increased flexibility in these antigen-binding loops. In contrast, the V_L_ chain exhibits generally lower RMSF values and less fluctuation across both CDRs, suggesting a more stable conformation.

**Extended Data Table 1 |:** Encoded amino acid sequences of B1.23.2 and HLA-B*44:05. Sequences of Fab V_H_, V_L_ of the B1.23.2 mouse IgG2a as well as the engineered chimeric mouse/human IgG1 V_H_C_H1_ molecules are presented in single-letter amino acid code. CDR1, CDR2, and CDR3 of both the V_H_ and V_L,_ as determined by the IMGT numbering system^93^, are color coded. The sequences of the HLA-B*44:05 heavy (A) and light (B, β_2_m) chains, as well as the antigenic peptide derived from HLA-DPA1*02:01_77–85,_ are shown. Epitope residues that contact with B1.23.2 are shown in firebrick color.

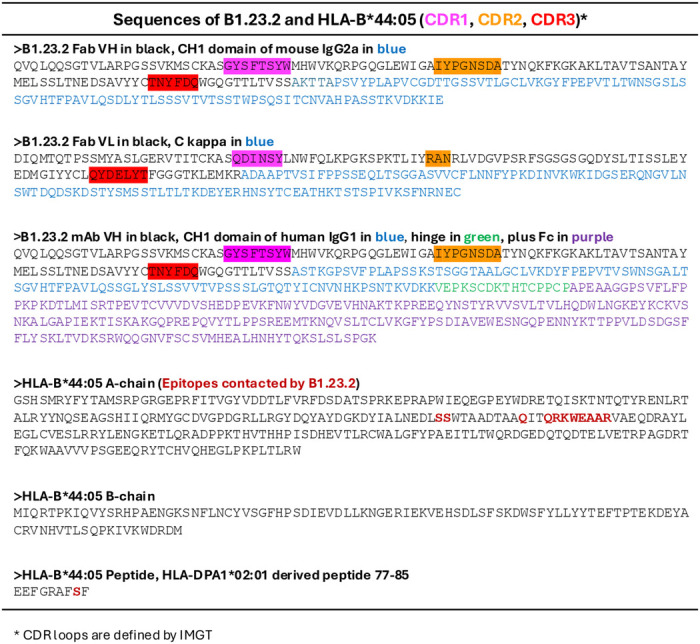

**Extended Data Table 2 |:** Contact tables. **a.** Fab B1.23.2 (9D73, 9D74, 8TQ6)**+**HLA-B*44:05. **b.** HLA-C*03:01+KIR2DL2 (1EFX) and **c.** HLA-B*57:01+KIR3DL1 (3VH8), (distances within 4.0 Å). Contact distances were calculated with PDBsum^94^. Only the closest contacts per residue are shown. In **a.** Fab chains are indicated by color (H, orange; L, magenta), and complementarity-determining regions (CDR loops) are noted.

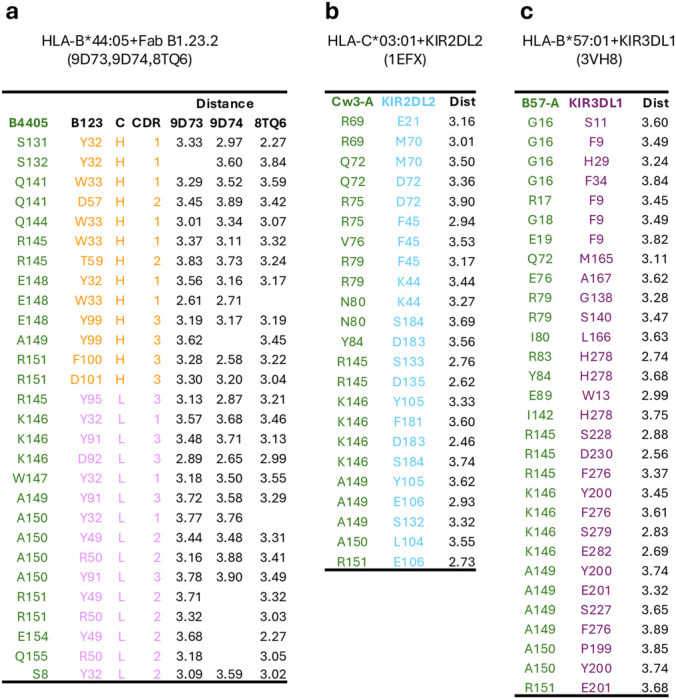

## Supplementary Material

This is a list of supplementary files associated with this preprint. Click to download.

- 8TQ6D1000276347valreportfullP1.pdf
- 9D74D1000285762valreportfullP1.pdf
- 9OA9D1000295033valreportfullP1.pdf
- 9D73D1000285761valreportfullP1.pdf

## Figures and Tables

**Table 1.** Cryo-EM data collection, refinement and validation statistics

	B1.23.2Ab+HLA-B*44:05	B1.23.2Fab+HLA-B*44:05	B1.23.2Ab-Fc
	(EMDB-46601)	(EMDB-46602)	(EMDB-70276)
	(PDB 9D73)	(PDB 9D74)	(PDB 9OA9)
**Data collection and processing**			
Magnification	130,000	130,000	130,000
Voltage (kV)	300	300	300
Electron exposure (e–/A^2^)	54.2	54.2	54.2
Defocus range (μm)	−0.7 to −2.0	−0.7 to −2.0	−0.7 to −2.0
Pixel size (Å)	0.83 (binned)	0.83 (binned)	0.83 (binned)
Symmetry imposed	C1	C1	C1
Initial particle images (no.)	1,525,615	1,447,993	1,447,993
Final particle images (no.)	359,876	406,922	251,084
Map resolution (Å)	3.02	3.31	3.44
FSC threshold	0.143	0.143	0.143
Map resolution range (Å)	3.02–3.50	3.31–3.90	3.40–3.70
**Refinement**			
Initial model used (PDB code)	8TQ6	8TQ6	Alphafold3
Model resolution (Å)	3.02	3.31	3.44
FSC threshold	0.143	0.143	0.143
Model resolution range (Å)	3.02–3.50	3.31–3.90	3.40–3.70
Map sharpening *B* factor (Å^2^)	0	0	0
Model composition			
Non-hydrogen atoms	6,311	6,017	3,676
Protein residues	807	798	550
Ligands	0	0	12
*B* factors (Å^2^)			
Protein	128.8	172.4	101.2
Ligand			
R.m.s. deviations			
Bond lengths (Å)	0.003	0.004	0.008
Bond angles (°)	0.618	0.682	1.302
Validation			
MolProbity score	2.50	2.71	1.64
Clashscore	11.65	16.56	51.75
Poor rotamers (%)	5.14	6.26	19.87
Ramachandran plot			
Favored (%)	94.48	94.40	68.90
Allowed (%)	5.52	5.60	28.19
Disallowed (%)	0.00	0.00	2.90

**Table 2.** X-ray crystallography Data collection and refinement statistics

	B1.23.2Fab+HLA-B*44:05
	(PDB-ID: 8TQ6)
**Data collection**	
Space group	P2_1_2_1_2_1_
Cell dimensions	
*a*, *b*, *c* (Å)	89.50, 92.84, 229.81
α, β, γ (°)	90.0, 90.0, 90.0
Resolution (Å)	49.30–3.20 (3.31–3.20)
*R*_sym_ or *R*_merge_	0.329 (1.346)
I /σ(*I)*	5.6 (1.5)
Completeness (%)	95.7 (94.2)
Redundancy	4.6 (4.3)
CC_1/2_	0.938 (0.435)
**Refinement**	
Resolution (Å)	49.30–3.20 (3.31–3.20)
No. reflections	31,179 (3,011)
*R*_work_ / *R*_free_	24.2/27.5 (29.9/32.2)
No. atoms	
Protein	12,516
Ligand/ion	0
Water	0
*B*-factors (Å^2^)	
Wilson Plot	45.1
Protein	47.7
Ligand/ion	0
Water	0
R.m.s. deviations	
Bond lengths (Å)	0.003
Bond angles (°)	0.72
Ramachandran	
favored (%)	92.9
allowed (%)	6.2
Outliers (%)	0.9

## Data Availability

The cryo-EM maps were deposited in the Electron Microscopy Data Bank under the accession IDs EMD-46601 (3.02 Å), EMD-46602 (3.31 Å) and EMD-70276 (3.44 Å), and the atomic coordinates were deposited in the PDB under the accession ID 9D73, 9D74, and 9OA9. X-ray crystal structure data and atomic coordinates were deposited in PDB under the accession ID 8TQ6.

## Abbreviations:

HLA: human leukocyte antigen
KIR: killer immunoglobulin-like receptor
LILR: leukocyte immunoglobulin-like receptor
MHC: major histocompatibility complex
mAb: monoclonal antibody
pMHC: peptide/MHC complex
SPR: surface plasmon resonance
TCR-T: cell receptor
NK: natural killer
NSG-IL15: NOD scid γ-immunodeficient mice, expressing a human IL-15 transgene
